# Does the Duration and Severity of Symptoms Have an Impact on Relief of Symptoms after Carpal Tunnel Release?

**DOI:** 10.1055/s-0038-1668552

**Published:** 2019-01-22

**Authors:** Mehreen Masud, Mamoon Rashid, Saleem Akhtar Malik, Muhommad Ibrahim Khan, Saad-ur-Rehman Sarwar

**Affiliations:** 1 Department of Plastic Surgery, Shifa International Hospital, Islamabad, Pakistan

**Keywords:** carpal tunnel release, carpal tunnel syndrome, severity of symptoms, duration of symptoms

## Abstract

**Rationale** Carpal tunnel syndrome (CTS) is the most frequently encountered compressive neuropathy of the upper limb. The treatment of CTS ranges from conservative management to carpal tunnel release. Many patients with misconception about the potential morbidity and with the hope of successful conservative treatment delay the surgical release of carpal tunnel. This delay results in reduced recovery of sensory and motor median nerve function.

**Objective** The aim of this study was to evaluate the influence of preoperative duration and severity of symptom on the outcome of carpal tunnel surgery.

**Method** It included 45 cases of CTS, all treated with limited access open carpal tunnel release. The duration of symptoms (i.e., pain, numbness, tingling, waking up at night because of pain/numbness, difficulty in grasping small objects, and their preoperative severity) was noted using Boston CTS questionnaire. To investigate the outcome, patients were divided into three groups based on their duration of symptoms.

**Result** Group1: The severity of symptoms was reduced to normal in a short period of time in patients who presented with duration of symptoms less than 6 months. Group 2: Patients in whom symptoms lasted for 6 to 12 months had reduced or delayed recovery of hand function as compared with first group. Group 3: Patients who had symptoms for more than 12 months had incomplete recovery of grip strength. Return to normal function took the longest time (median: 16 weeks) in this group.

**Conclusion** This study suggests that patients who present late have delayed/incomplete relief of symptoms after carpal tunnel release.

## Introduction

Carpal tunnel syndrome (CTS) is the most frequently encountered compressive neuropathy of the upper limb. The American Academy of Orthopedic Surgeons defines CTS as “A symptomatic compression neuropathy of the median nerve at the level of wrist.” The symptoms include numbness of medial three digits of the hand, reduced manual strength, tingling sensation, nocturnal numbness/pain, and difficulty in gripping small objects. 1

CTS accounts for 90% of all nerve entrapment syndromes. Its prevalence in general population is reported as 3.8%, with an incidence of 276:100,000 per year. 1 Its release is the most commonly performed elective hand operation with 0.82 to 2.87 operations per 1,000 individuals done each year in the United States alone. 2 Various studies have been done to evaluate accurate diagnostic criteria and the selection of treatment modality to predict a better outcome for the patient, which not only improve the patient's general well-being but also shrink the annual costs to society. 3 4

The treatment of CTS ranges from conservative management to carpal tunnel release. The conservative care includes splinting, vitamin B6, nonsteroidal anti-inflammatory agents, and local corticosteroid injections. The options for CTS release can be standard open carpal ligament release, limited access open release, limited access device-assisted release, and two-port and single-port endoscopic release. 4 Endoscopic release results in decreased postoperative pain, reduced scar length, and early return to work. However, when hand function is assessed for return of grip strength, dexterity, and sensation, no noteworthy superiority of one technique can be documented. 5 Transverse carpal ligament release remains the mainstay of management and is important in early recovery of nerve function. 6 Many patients with apprehensions about the morbidity and the hope of successful conservative treatment delay the surgery 7 and can have reduced recovery of sensory and motor function. 8 9 Thus, to reasonably predict the outcome of such surgery, the influence of duration 10 and preoperative severity of symptoms 11 on the outcome of carpal tunnel surgery should be evaluated. The outcome of carpal tunnel release is assessed in terms of return of hand function and relief of symptoms. The purpose of this study is to investigate the afore mentioned relationship.

## Patients and Methods

**Fig. 1:**
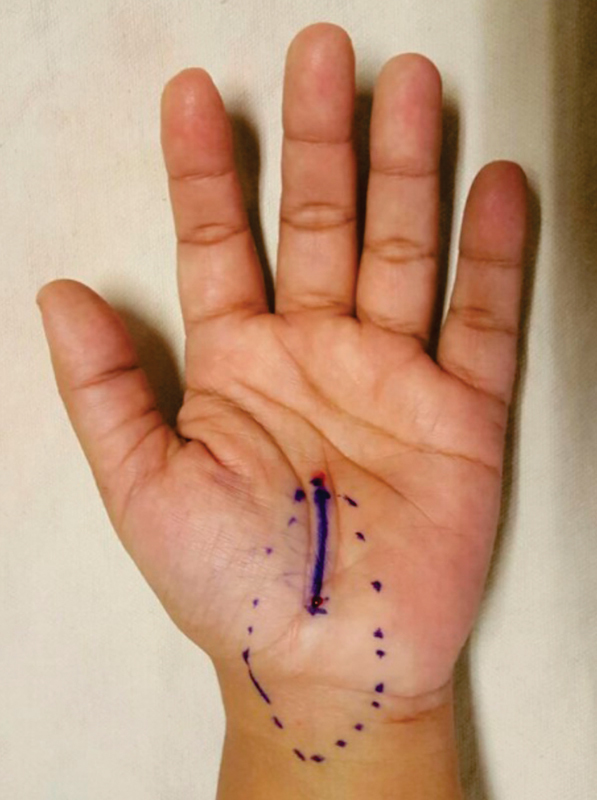
Preoperative marking of carpal tunnel syndrome.

**Fig. 2:**
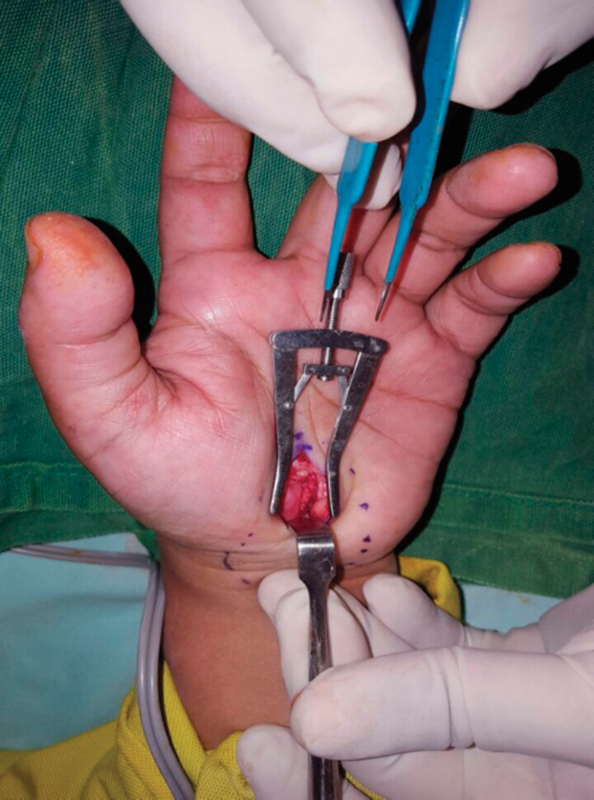
Exposure of median nerve after release of transverse ligament.

This prospective study was performed at the Department of Plastic Surgery from January 2015 to January 2017. Diagnosis of CTS was established using clinical criteria (CTS-6), described by Graham et al (a pub), 4 outlined in Table 1 . It was confirmed by nerve conduction studies. All patients were treated by limited incision release under local anesthesia ( Figs. 1 and 2 ). Patients with cervical neuropathy, polyneuropathy, rheumatoid arthritis, wrist fractures, osteoarthritis of wrists, space-occupying lesion in carpal tunnel, and ulnar nerve entrapment at Guyon's canal were excluded from the study.

**Table 1:** CTS-6 diagnostic clinical criteria for CTS 4

**1.**	Numbness and tingling in the median nerve distribution
**2.**	Nocturnal numbness
**3.**	Weakness and/or atrophy of the thenar musculature
**4.**	Tinel's sign
**5.**	Phalen's test
**6.**	Loss of two-point discrimination

Abbreviation: CTS, carpal tunnel syndrome.

The duration of symptoms (i.e., pain, numbness, tingling, waking up at night because of pain/numbness, difficulty in grasping small objects, and their preoperative severity) was noted using Boston CTS questionnaire (validated questionnaire). 5 To investigate the outcome, our patients were divided into three groups—Group 1: patients in whom the symptoms lasted for less than 6 months; Group 2: patients who had symptoms for 6 to 12 months; and Group 3: patients in whom the duration of symptoms spanned more than 12 months.

Postoperatively, patients were followed up at 1 week, 2 months, and 4 months and a final assessment was performed at 6 months. Time taken for postoperative relief of symptoms and severity abatement was documented based on the Boston questionnaire.

## Statistics

Bivariate and multivariate analyses of data were done by Statistical Software for Social Sciences (SPSS version 21). Qualitative data were expressed as frequencies and percentages, whereas quantitative data were expressed as median ± interquartile range (IQR). Normality of data has been checked by Kolmogorov–Simonov test. Spearman's correlation established the positive and negative correlation of the variables. Wilcoxon's sign-rank test was used to determine the difference in the variables before and after the surgery. Kruskal–Wallis one-way analysis of variance (ANOVA) was used to determine the difference in groups considering all statistical tests significant with *p* -value < 0.05.

## Results

**Fig. 3:**
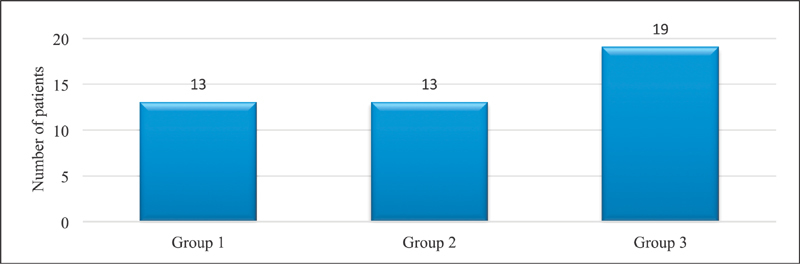
Distribution of patients in each group.

A total of 45 patients were treated at our department from January 2015 to 2017. Out of which, 40 were included in the study (88% of the total patients, of whom 89% were female). Of the 40 patients who underwent surgery, 5 patients (11%) had bilateral CTS and all were analyzed for each hand separately. A total of 45 hands were followed up over a period of 24 weeks. Their distribution in each group is shown in Fig. 3 .

### Group 1 (Duration of Symptoms Less than 6 Months)

Thirteen patients (28% of all patients) presented in this group with median age of 50 years (IQR: 27 years), 84% female and 15% male. Among these patients, the foremost symptom was pain and numbness in the distribution of the median nerve.

**Fig. 4:**
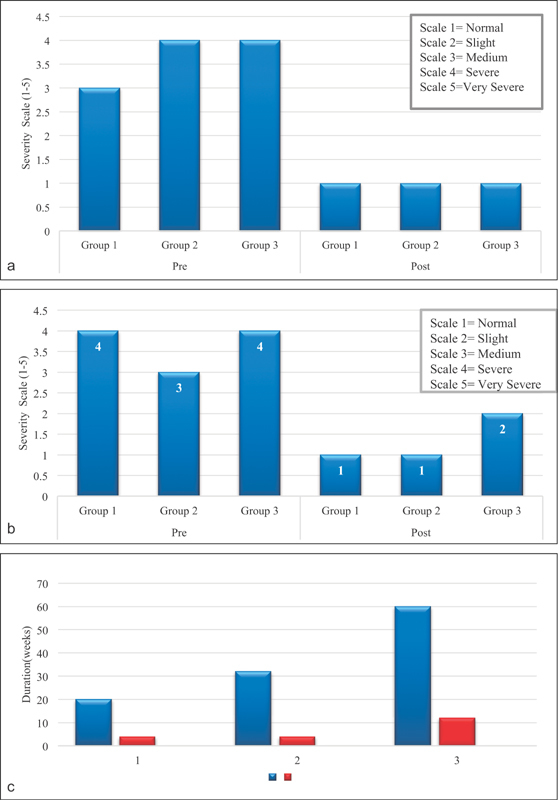
( **a** ) Comparison of preoperative and postoperative severity of pain at daytime. ( **b** ) Comparison of preoperative and postoperative severity of pain at night. ( **c** ) Median duration of pain and its recovery in each group.

The average preoperative severity of pain during the day was graded as 3 ( *n* = 5) on Boston questionnaire and the postoperative severity was documented as 1 (1 was taken as normal; Fig. 4a ). Similarly, before surgical intervention, the average severity of pain at night was graded as 4 ( *n* = 8) and postoperatively it was reported as 1 ( Fig. 4b ). The median duration of pain in the preoperative period was 20 weeks (IQR: 15 weeks). It was relieved in a duration of 4 weeks (IQR: 8 weeks) after the operation ( Fig. 4c ).

**Fig. 5:**
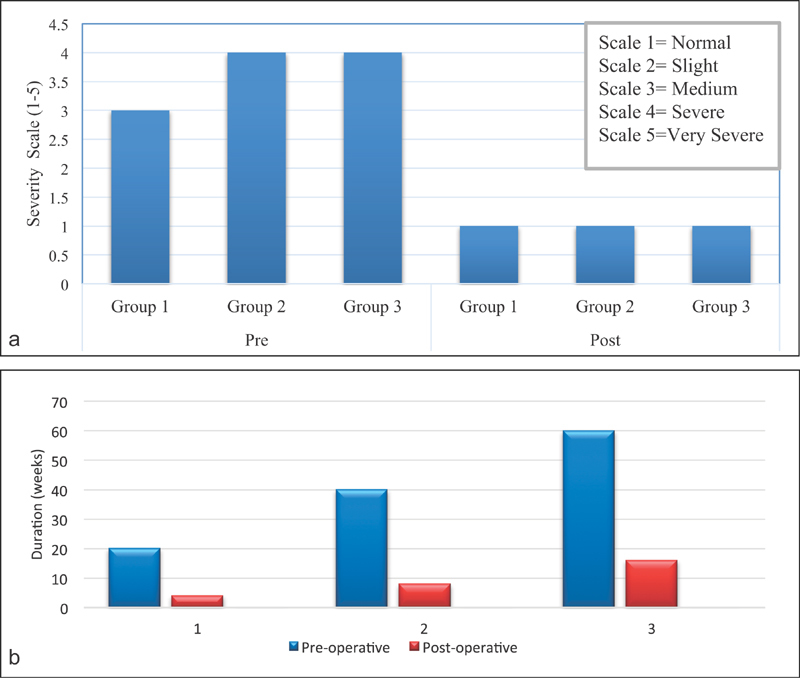
( **a** ) Comparison of preoperative and postoperative severity of numbness in each group. ( **b** ) Comparison of median duration of numbness and its recovery in three groups.

**Fig. 6:**
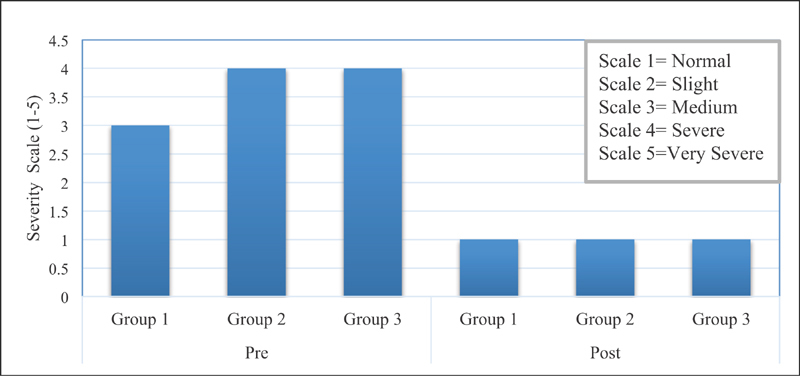
Comparison of preoperative and postoperative severity of tingling in each group.

**Fig. 7:**
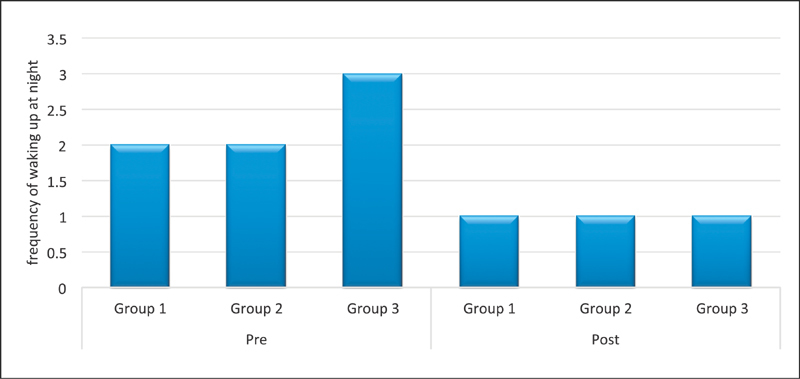
Comparison of waking up at night before and after surgery.

Similarly, the preoperative severity of numbness graded as 3 was reduced to normal (grade 1) in a short span of 4 weeks ( Fig. 5a , b ). Similar results were obtained when preoperative and postoperative severity of tingling and frequency of waking up at night because of pain and numbness were compared ( Figs. 6 and 7 ).

The presurgery weakness of grip strength was reported as medium (grade 3), recorded on a scale of 1 to 5. It returned to normal (grade 1) within 8 weeks (IQR: 12; Figs. 8a , b ). The median return-to-normal function took 8 weeks (IQR: 12 weeks) in this group ( Fig. 9 ). Thus, the severity of symptoms was reduced to normal in a short span of time in this group.

**Fig. 8:**
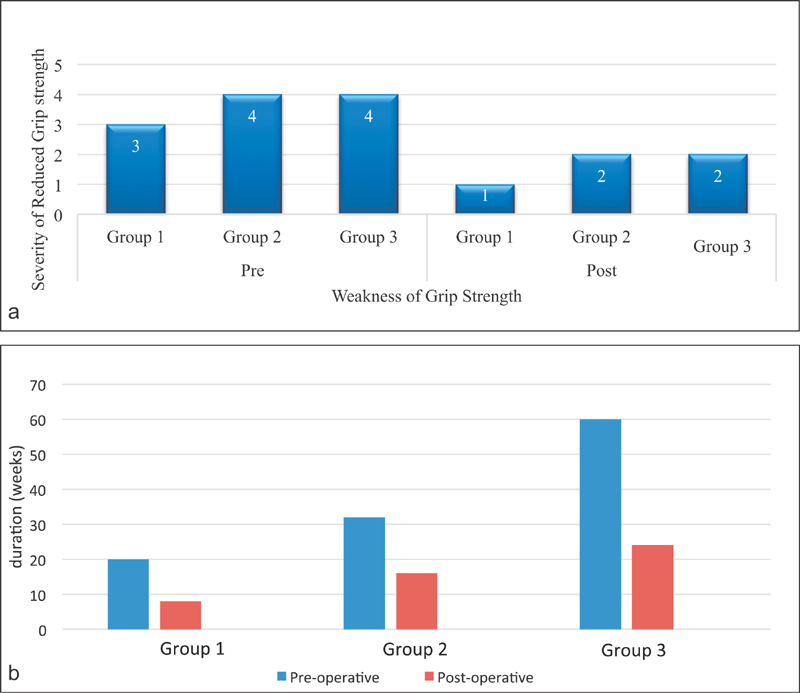
( **a** ) Comparison of preoperative and postoperative weakness *of grip* strength. ( **b** ) Comparison of preoperative duration and postoperative recovery period of reduced grip strength in each group.

**Fig. 9:**
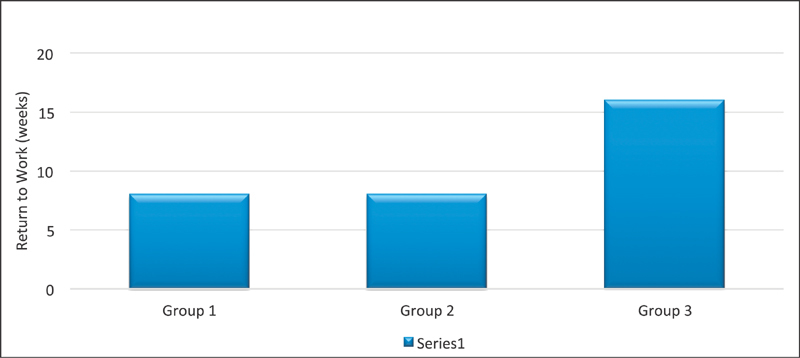
Comparison of return to work in each group.

### Group 2 (Duration of Symptoms 6–12 Months)

The second group included 13 patients ( *n* = 19, 28% of the total patients) who experienced symptoms for 6 to 12 months. The median age in this group was 54 years (IQR: 25), 76% of whom were female. In our study, these patients were pain free after a median of 4 weeks, IQR of 8 weeks ( Fig. 4c ). The results of this group were similar to the first group in terms of reduction of severity of pain, numbness, and tingling ( Figs. 4a , b ; Fig. 5a , Fig. 6 , respectively). The weakness of grip strength was rated as 4 (severe) preoperatively. In our work, the return of normal grip strength was not achieved in patients with duration of symptom longer than 6 months ( Fig. 8a ). The median return to work in this group was noted as 8 weeks, IQR of 8 weeks ( Fig. 9 ).

### Group 3 (Duration of Symptoms More than 12 Months)

Patients whose symptoms lasted for more than 12 months ( *n* = 19, 100% female) were included in the third group (median age: 50 years, IQR: 13 years). The mean duration of pain was 60 weeks and its complete relief was achieved within 12 weeks (IQR: 23 weeks). A comparison of preoperative severity of numbness and its recovery in each group ( Fig. 5a ) showed that although patients with delayed presentation took longer to recover, complete recovery was achieved. Preoperative weakness of grip strength was rated as 4 and postoperative was 2 and median duration of return of grip strength was 24 weeks (IQR: 23 weeks). Recovery of grip strength was incomplete in this group and return to normal function took the longest time (median: 16 weeks/IQR: 20 weeks).

The incidence of preoperative and postoperative duration and severity of pain in day time, severity of pain at night, numbness, tingling, waking up at night because of pain, and grip strength were calculated and are graphically shown in Figs. 3 4 5 6 7 8 . The duration of return to work was also compared in these groups ( Fig. 9 ).

Patients with duration of pain more than 12 months underwent CTS release and their symptoms were relieved after a longer period of time. However, once recovery was achieved, either minimal or no pain was present in all group, as shown graphically in Figs. 4a , c .

A comparison of preoperative severity of numbness and its recovery in each group is depicted in Fig. 5a . The severity was measured on scale 1 to 5. The median for recovery of numbness was similar in each group. The comparison of Figs. 5a , b indicates that although patients with delayed presentation require longer duration to recover, full recovery was achieved.

In this table, preoperative and postoperative severity of tingling was recorded using scales 1 to 5. The median value for postoperative recovery of tingling was 1 in all groups which was labeled as slight on the scale indicating it recovery to almost normal.

The preoperative weakness and postoperative recovery of reduced grip strength was graded 1 to 5, with grade 1 labeled as grasping without any difficulty, grade 2 as little difficulties in grasping, grade 3 as moderate difficulty, grade 4 as very difficult to grasp, and grade 5 as unable to grasp. The preoperative severity of grip strength was rated as 4 by the patients who reported duration of symptoms more than 6 months and it was never recovered to its normal status till the last follow-up in these groups.

Fig. 8b shows a preoperative duration of reduced grip strength and its duration of recovery in each group. Patients with longer duration of reduced grip strength require more time for its recovery postoperatively.

The median functional status was delayed by 16 weeks in patient with late presentation of symptoms.

The Wilcoxon signed-rank test shows a statistically significant difference in duration of presurgery and postsurgery group with respect to pain, numbness, grip strength, and tingling with *p* < 0.05. Kruskal–Wallis one-way analysis of variance (ANOVA) was used to analyze duration of relief of numbness among the three groups. It shows that there is a statistically significant difference in duration of relief of numbness in the three groups with *p* < 0.05. Spearman's correlation shows the positive correlation in preoperative and postoperative duration of variables, which shows those who present late have more chances of delayed relief ( Figs. 3 4 5 6 7 ).

## Discussion

With increasing life expectancy, the incidence of CTS is escalating as 3.5% of males and 11% of females are anticipated to develop this compression neuropathy each year. 12 This increasing magnitude of the disease demands an early effective treatment.

The open release of flexor retinaculum, published by Phalen et al (a pub) in 1950, has been the standard treatment for the CTS for decades. 13 Because of the concerns related to postoperative morbidity, some studies endorse the use of steroid injections or nonsteroidal anti-inflammatory drugs, for the management of CTS. 10 However, when analyzed by Brown et al (a pub), 10 these studies showed that conservative management may be useful only in patients who present with early mild symptoms only. 10

Endoscopic carpal tunnel release was described by Okutsu et al (a pub) in 1989. Although this operation appears to offer potential advantages, when compared with open release in terms of return of hand function, no significant superiority of one technique over the other can be documented. 5 13 This led us to investigate the factors other than the type of procedure that might influence the outcome for the patient.

In this study, 40 patients underwent open carpal tunnel release on 45 hands using a limited exposure technique ( Fig. 3 ). The median age of the patients was 50 years which composed of 88% females and 11% males. Cozen et al have shown a similar trend, noting a preponderance of women in the population affected by CTS. 14 She reported 63% of females versus 36% of males to be affected by CTS in her study.

Preoperative and postoperative symptom severity score of pain, numbness, tingling, waking up at night, reduced grip strength, and their duration of recovery were assessed individually in our study. They are discussed in detail later.

### Pain

The preoperative severity of pain was recorded separately during day time and at night in our study. It was found to be highest in patients with duration of symptoms lasting for more than 12 months. However, postoperatively, daytime pain was reduced to normal in all groups, while at night it was reported to be slightly more (scale 2) in patients who had symptoms for more than 12 months. These results were similar to that of Burke et al 11 who stated that the patients with highest preoperative symptom severity had the greatest improvement in severity score, although they did not reach the same final scores as in patients with mild symptom. Burke et al did not record the pain scores separately during day time and at night. When our results of the aforementioned times are reviewed simultaneously, the results were similar to that of Burke et al. 11 The duration of recovery of pain was longest in patients of group 3, similar to the study of Eisenhardt et al. They reported a recovery period of 25 days in patients who suffered from symptoms of CTS for more than 12 months compared with 16 days of recovery in patients with duration of symptoms less than 12 months. 5

### Sensory Abnormalities

The pretreatment severity and duration of recovery of numbness and tingling were highest in patients with delayed presentation, yet complete recovery was achieved in all groups. These results are akin to the work presented by Cozen et al. 14 They reported that patients with thenar atrophy due to prolong duration of symptoms scored 0.35 (95% confidence interval) points higher in the Boston Questionnaire score than patients without it. 14

The frequency of waking up at night due to pain and paresthesia was highest in patients who suffered more severe and prolonged symptoms; however, it was reduced significantly in all groups postoperatively. This corresponds well to the study of Eisenhardt et al, who reported that the duration of paresthesia did not had significant influence on the outcome of the operation. 5

### Hand Function

Results of our study indicate that patients who presented with a prolonged duration of symptoms require a longer period to recover, especially in terms of hand function and return to work. This was akin to the work of Eisenhardt et al who showed a strong relationship between duration of symptoms and return to vocational activities and normal hand function ( *p* < 0.001). 5 Patients with prolonged duration of reduced grip strength require longer postoperative duration to recover the strength and the recovery is also incomplete. Brown et al (a pub) 10 have suggested that this may be due to progressive median nerve damage in long-standing CTS.

### Complications

No critical complications, for instance laceration of nerve, vessels, or tendons, were encountered in our work. This is attributable to the fact that we used open access technique for carpal tunnel release in all our cases. Zou et al 13 compared endoscopic versus open carpal tunnel release for idiopathic CTS and stated that the incidence of nerve injury with open release is comparatively low (confidence interval of 0.98 vs. 5.77). Moreover, all the operative work was done by senior author (M.R.) of the study who has 25 years of experience with this technique; so, we encountered no major complications in our work. The other complications of CT release like postoperative pain, reduced grip strength, and delayed return to vocational activities were documented as variables in this study. However, pillar pain and hypertrophic scaring were not analyzed in our work.

## Conclusion

On the basis of our study, we strongly recommend early release of the transverse carpal ligament, which can potentially avoid permanent median nerve damage and thus allow for early rehabilitation of the patient. 5 A low complication rate and the fact that patients with longer duration of symptoms had delayed and incomplete recovery strongly suggests that early intervention should be encouraged. Although in our study the return to work was similar in patients with symptoms less than 6 months and those with symptoms lasting 6 to 12 months, we suggest early surgical procedure as the patients remain partly disabled in the hope of recovery by conservative treatment. Furthermore, our study shows that there is statistically significant improvement of median symptom-severity score in the patients who presented early and were treated early surgically.
